# Supplementary material for: Knowledge of acute stroke management and the predictors among Malaysian healthcare professionals
Source: PeerJ. 2022 Apr 20;10:e13310. doi: 10.7717/peerj.13310 (PMC9034705; doi:10.7717/peerj.13310)
Supplement: Supplemental Information 1 [file peerj-10-13310-s001.docx]

**Table S1: Classification of level of knowledge.**

| **Levels of knowledge** | **Likert scale responses^#^**  **(Cutoff point = 3.5)** | **Range of scores calculation** | **Overall**  **(29 items)** | **GSK**  **(10 items)** | **HSM**  **(9 items)** | **ASM**  **(10 items)** |
| --- | --- | --- | --- | --- | --- | --- |
| Good | 4 & 5 | ≥ (Total number of items× 3.5) | 101.5-145 | 35-50 | 31.5-45 | 35-50 |
| Poor | 1, 2 & 3 | < (Total number of items× 3.5) | 29-101.5 | 10-35 | 9-31.5 | 10-35 |

^#^After reverse coding done for negative answers as desired response.
