# Supplementary material for: Knowledge of acute stroke management and the predictors among Malaysian healthcare professionals
Source: PeerJ. 2022 Apr 20;10:e13310. doi: 10.7717/peerj.13310 (PMC9034705; doi:10.7717/peerj.13310)
Supplement: Supplemental Information 2 [file peerj-10-13310-s002.docx]

**Table S2: Participants' responses and scores for the ASMaQ.**

| **Questions** | **Responses** | | | **Scores** |
| --- | --- | --- | --- | --- |
|  | **Strongly agree/ Agree**  ***n* (%)** | **Neutral**  ***n* (%)** | **Disagree/ Strongly disagree**  ***n* (%)** | **Median (IQR)** |
| **General Stroke Knowledge** [Mean (SD) = 39.18 (3.40)] |  | | | 40 (5) |
| 1. Acute confusion may be a sign of stroke. | 563 (89.8) | 38 (6.1) | 26 (4.1) | 5 (1) |
| 1. Hypoglycaemia can mimic acute stroke. | 577 (92.0) | 30 (4.8) | 20 (3.2) | 5 (1) |
| 1. Stroke can present with visual disturbances. | 593 (94.6) | 20 (3.2) | 14 (2.2) | 5 (1) |
| 1. Stroke patients can present with limb numbness. | 591 (94.3) | 24 (3.8) | 12 (1.9) | 5 (0) |
| 1. Unsteadiness of gait can be a sign of stroke. | 605 (96.5) | 18 (2.9) | 4 (0.6) | 5 (0) |
| 1. Acute stroke can present with reduced level of consciousness. | 608 (97.0) | 14 (2.2) | 5 (0.8) | 5 (0) |
| 1. The Glasgow Coma Scale (GCS) is a tool to assess level of consciousness. | 604 (96.3) | 11 (1.8) | 12 (1.9) | 5 (0) |
| 1. *A full neurological examination must be performed immediately in patients presenting acutely with symptoms suggestive of stroke.** | 562 (89.6) | 42 (6.7) | 23 (3.7) | 1 (1) |
| 1. *High blood pressure must be lowered to normal values in acute stroke.** | 92 (14.7) | 68 (10.8) | 467 (74.5) | 4 (2) |
| 1. *Acute stroke management education should be conducted regularly for healthcare professionals.** | 615 (98.1) | 11 (1.8) | 1 (0.2) | 1 (0) |
| **Hyperacute Stroke Management** [Mean (SD) = 32.43 (3.67)] |  | | | 32 (5) |
| 1. *Stroke is a medical emergency only within 4.5 hours of stroke onset.** | 347 (55.3) | 92 (14.7) | 188 (30.0) | 2 (3) |
| 1. All acute stroke patients must undergo a brain CT immediately. | 569 (90.7) | 33 (5.3) | 25 (4.0) | 5 (1) |
| 1. All suspected stroke patients must be referred to the neurology team immediately. | 463 (73.8) | 115 (18.3) | 49 (7.8) | 4 (2) |
| 1. The earlier the treatment, the better the outcome of acute stroke. | 614 (97.9) | 11 (1.8) | 2 (0.3) | 5 (0) |
| 1. Thrombolysis therapy is given intravenously to breakdown clots. | 577 (92.0) | 25 (4.0) | 25 (4.0) | 5 (1) |
| 1. My hospital is equipped with thrombolysis treatment. | 336 (53.6) | 85 (13.6) | 206 (32.9) | 4 (3) |
| 1. *Coagulation profile must be screened before thrombolysis.** | 464 (74.0) | 86 (13.7) | 77 (12.3) | 2 (2) |
| 1. *All acute stroke patients must have a 12 leads ECG before thrombolysis.** | 510 (81.3) | 74 (11.8) | 43 (6.9) | 1 (1) |
| 1. Intracranial haemorrhage is a contraindication for thrombolysis therapy. | 598 (95.4) | 15 (2.4) | 14 (2.2) | 5 (0) |
| **Advanced Stroke Management** [Mean (SD) = 36.32 (5.35)] |  | | | 36 (8) |
| 1. Are you able to detect symptoms of acute stroke?   {Very likely/Likely; Neutral; Unlikely/Very unlikely} | 584 (93.1) | 37 (5.9) | 6 (1.0) | 4 (1) |
| 1. Are you familiar with FAST (Face, Arm, Speech, Time)?   {Very familiar/Familiar; Neutral; Not familiar/Never heard of it} | 475 (75.8) | 64 (10.2) | 88 (14.0) | 4 (1) |
| 1. How would you rate your knowledge on acute stroke management?   {Very good/Good; Neutral; Poor/Very poor} | 312 (49.8) | 279 (44.5) | 36 (5.7) | 3 (1) |
| 1. Are you aware of mechanical thrombectomy treatment for stroke?   {Well aware/Aware; Neutral; Not familiar/Never heard of it} | 383 (61.1) | 103 (16.4) | 141 (22.5) | 4 (2) |
| 1. Mechanical thrombectomy is administered for clot removal in acute stroke. | 432 (68.9) | 168 (26.8) | 27 (4.3) | 4 (2) |
| 1. My hospital is equipped with mechanical thrombectomy service. | 93 (14.8) | 118 (18.8) | 416 (66.3) | 2 (2) |
| 1. Acute stroke symptoms can be potentially reversed with administration of thrombolysis or with mechanical thrombectomy. | 524 (83.6) | 88 (14.0) | 15 (2.4) | 4 (1) |
| 1. Mechanical thrombectomy can be performed after thrombolysis therapy. | 238 (38.0) | 259 (41.3) | 130 (20.7) | 3 (1) |
| 1. Thrombolysis and mechanical thrombectomy can only be administered within a therapeutic window. | 488 (77.8) | 108 (17.2) | 31 (4.9) | 4 (1) |
| 1. *Wake up strokes are not eligible for thrombolysis nor mechanical thrombectomy.** | 170 (27.1) | 289 (46.1) | 168 (26.8) | 3 (2) |
| **Total scores** [Mean (SD) = 107.20 (9.29)] |  | | | 107 (13) |

*Asterisk sign and italic sentence denotes negative answer as desired response.
