# Supplementary material for: Knowledge of acute stroke management and the predictors among Malaysian healthcare professionals
Source: PeerJ. 2022 Apr 20;10:e13310. doi: 10.7717/peerj.13310 (PMC9034705; doi:10.7717/peerj.13310)
Supplement: Supplemental Information 3 [file peerj-10-13310-s003.pdf]

Figure S1: Area under the ROC curve - shows the model's ability to discriminate between good and poor knowledge for overall knowledge on stroke.

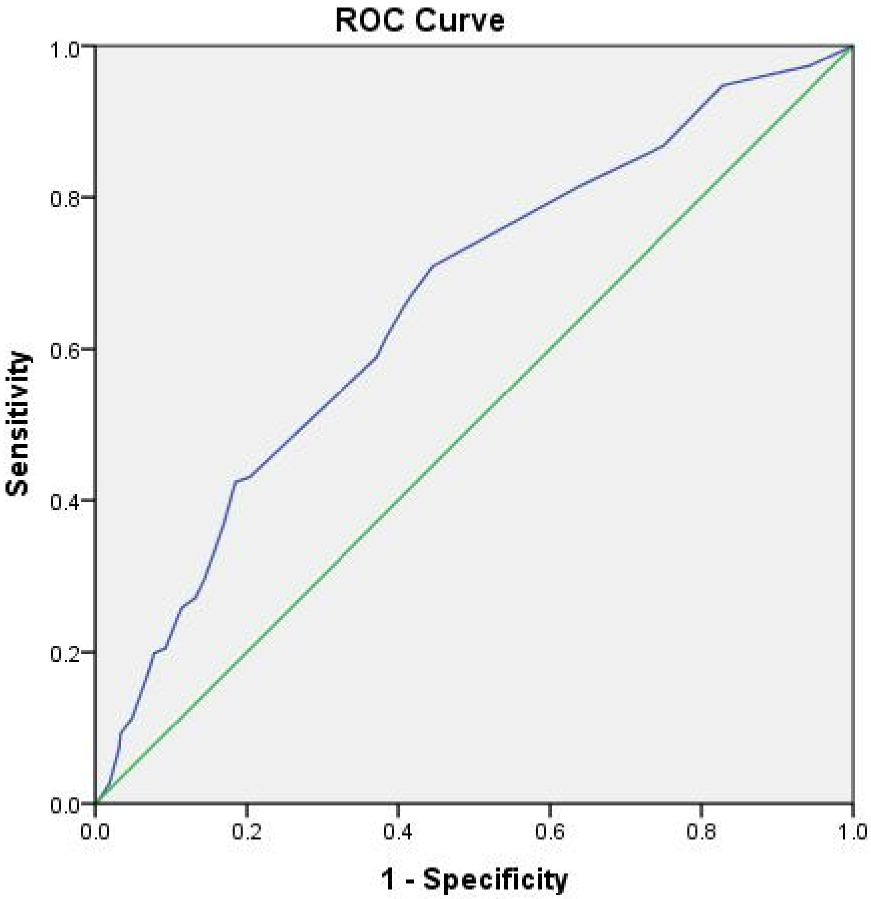

Diagonal segments are produced by ties.

Area Under the Curve

Test Result Variable(s): Predicted probability

Area	Std. Error[a]	Asymptotic Sig.[b]	Asymptotic 95% Confidence Interval	
			Lower Bound	Upper Bound
.658	.025	.000	.608	.708

The test result variable(s): Predicted probability has at least one tie between the positive actual state group and the negative actual state group. Statistics may be biased.

a. Under the nonparametric assumption

b. Null hypothesis: true area = 0.5
